# Impact of urate-lowering drugs on the progression and recovery from chronic kidney disease among gout patients

**DOI:** 10.1186/s13075-019-1993-9

**Published:** 2019-09-18

**Authors:** Ting-Ting Chung, Kuang-Hui Yu, Chang-Fu Kuo, Shue-Fen Luo, Meng-Jiun Chiou, Wen-Ching Lan, Jung-Sheng Chen, Wen-Yi Tseng, Ao-Ho Hsieh, Lian-Chin Wang

**Affiliations:** 1 Division of Rheumatology, Allergy and Immunology, Chang Gung Memorial Hospital, No. 5, Fu-Hsing Street, Taoyuan, 333 Taiwan; 2 Center for Artificial Intelligence in Medicine, Chang Gung Memorial Hospital, Taoyuan, Taiwan; 3 Center for Big Data Analytics and Statistics, Chang Gung Memorial Hospital, Taoyuan, Taiwan

**Keywords:** Gout, Urate-lowering drug, Chronic kidney disease, Renal function, Allopurinol, Febuxostat, Uricosuric agents

## Abstract

**Background:**

This study investigates the association between exposure to urate-lowering drugs (ULDs) and progression and recovery from chronic kidney disease (CKD).

**Methods:**

We identified 5860 incident gout patients at Chang Gung Memorial Hospital from 2012 to 2015. Propensity score (PS)-weighted Cox proportional hazards model was used to estimate hazard ratios (HRs) for CKD progression and improvement. A separate analysis was conducted to assess the HR for CKD progression and CKD recovery among those with worsening CKD.

**Results:**

The incidence of CKD progression among allopurinol, febuxostat and uricosuric agent users were 1.98, 1.88 and 1.64 per 1000 person-days. Compared with allopurinol users, the PS-weighted HR (95% confidence intervals [CIs]) was 1.77 (0.85–1.76) for febuxostat users and 1.37 (0.71–1.37) for uricosuric agent users for CKD progression and 1.43 (1.26–1.62) for febuxostat users and 1.00 (0.88–1.14) for uricosuric agent users for CKD improvement. Compared to allopurinol users, the HRs for CKD progression were 1.14 (0.80–1.66) for febuxostat users and 0.92 (0.67–1.31) for uricosuric agent users. Among 741 patients who had CKD progression, the incidence of CKD recovery was 1.33, 6.21 and 3.53 per 1000 person-days for allopurinol, febuxostat and uricosuric agent users. The HRs (95% CIs) for recovery in febuxostat and uricosuric agent users were 2.17 (1.40–3.47) and 1.80 (1.20–2.83) compared to allopurinol users.

**Conclusions:**

CKD progression and recovery are common in gout patients using ULDs. Febuxostat and benzbromarone were associated with a similar risk of CKD progression with allopurinol, which has a poorer recovery compared with other ULDs.

## Background

Gout is an inflammatory arthritis with an increasing prevalence worldwide [1]. It is associated with multiple comorbidities including chronic kidney disease (CKD) [2]. Chronic hyperuricaemia and subsequent crystal deposition in renal parenchyma have been found to induce a reduction in renal blood flow, glomerulosclerosis [3], urate nephropathy and urolithiasis [4]. All of these conditions can negatively affect renal function and lead to acute kidney injury or CKD. To potentially lower this risk of CKD, urate-lowering drugs (ULDs) which normalize serum uric acid have been used, including xanthine oxidase inhibitors (allopurinol and febuxostat) and uricosuric agents (benzbromarone, probenecid, sulfinpyrazone and lesinurad), the two main classes of ULDs.

However, the results of the effect of ULDs on renal function have been inconsistent. Unlike uricosuric agents which enhance renal urate clearance and potentially aggravate the risk of urolithiasis [5], allopurinol has been found to reduce the risk of CKD progression [6]. The renal safety of febuxostat has been demonstrated in a few studies [7, 8]. Recently, a clinical trial enrolling 96 patients with moderate to severe renal impairment found that 12 months of febuxostat treatment did not seem to influence changes in serum creatinine [9]. In addition, the renal safety of benzbromarone in patients with CKD was documented in a small study comprising 48 patients [10]. However, in real-world practice, gout patients have poor compliance, are at high risk of CKD and are frequently given nephrotoxic agents such as non-steroidal anti-inflammatory drugs (NSAIDs).

The association between exposure to different classes of ULDs and CKD progression and recovery has not been investigated. Therefore, we performed this cohort study using data from Chang Gung Memorial Hospital, the largest medical centre in Taiwan, including patients with a new diagnosis of gout and compared the risk of CKD progression and recovery between patients exposed to different classes of ULDs. The main objectives were to investigate whether the use of ULDs in gout patients would influence the CKD progression by one stage while controlling for potential confounders, and to explore whether the observed effect was specific to a particular drug class.

## Methods

The incident gout cohort was identified from the Chang Gung Research Database, which contains the electronic health records of all patients who visited any one of the following seven Chang Gung Memorial Hospitals: Keelung, Taipei, Linko (headquarters), Taoyuan, Yunlin, Chiayi and Kaohsiung. Chang Gung Memorial Hospital is the biggest private medical center in Taiwan and services approximately 7% of all patient visits in Taiwan. Valid internal patient record linkage is achieved by using unique patient identifiers. To ensure confidentiality, these identifiers are encrypted before the data is released to researchers. This study only used anonymized and nontraceable data, and therefore, the need for patient consent was waived.

### Study design

We conducted this cohort study to examine the risk of CKD progression among patients with gout associated with different ULDs. People aged ≥ 18 years who had a diagnosis of gout and at least one prescription of a ULD between 2012 and 2015 were identified, and their electronic health record data were collected from their first visit to Chang Gung Memorial Hospital (as early as January 2001) through December 2016 with information regarding patient demographics, outpatient and inpatient diagnoses, medications, laboratory results, operations and procedures. The patient classification is based on the American Rheumatism Association preliminary classification criteria for acute gout [11].

**Fig. 1:**
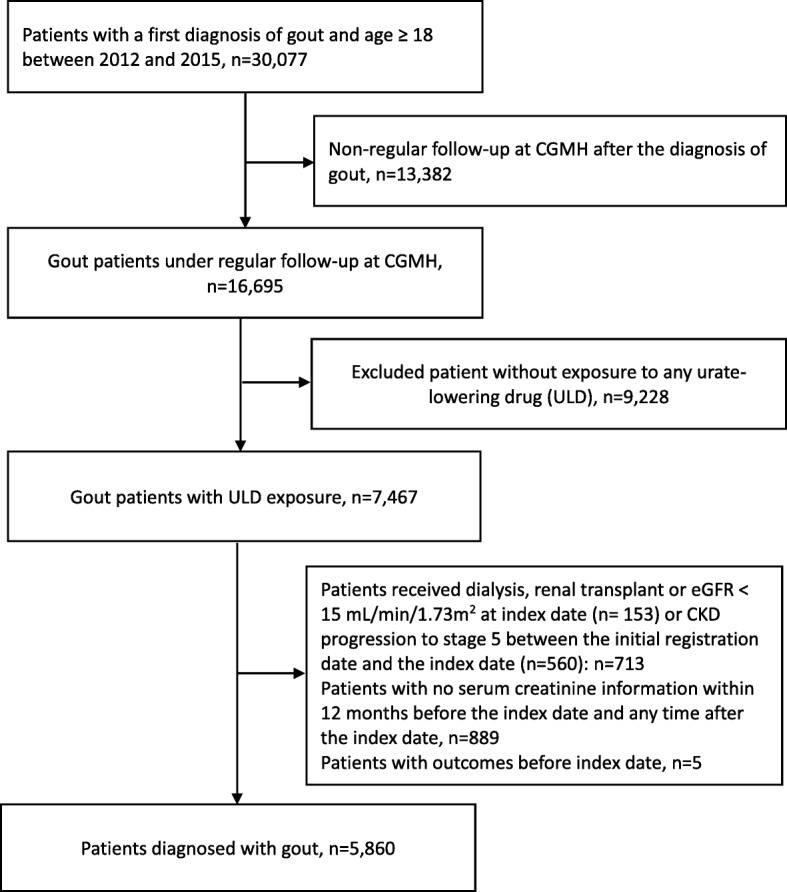
Flow chart of the study cohort

The prescriptions of ULDs (febuxostat, allopurinol and uricosuric agents including benzbromarone, sulfinpyrazone and probenecid) were identified. The patients were classified according to their first ULD (allopurinol users, febuxostat users and uricosuric agent users). The patients prescribed with two or more ULDs were excluded from the analysis. We identified the initial registration date to the Chang Gung Memorial Hospital and index date. The index date was defined as the first date of ULD exposure. Those with CKD stage 5 at the index date or CKD stage progression to stage 5 any time between the initial registration date and the index date and those without data on serum creatinine level during the study period were also excluded. This is to ensure the CKD progression is related to exposure to ULD. The flow chart of cohort selection is shown in Fig. 1. The patients were followed from the index date and censored at the earliest date of the first progression of CKD by one stage, switch to a different ULD, death or the end date of the study (30 June 2016). We then identified those with CKD progression by one stage and followed and censored them at the earliest date of the first recovery of CKD by one stage, death or the end date of the study (30 June 2016).

The CKD stage of each patient was determined at the first ULD prescription (index date). The CKD stage was defined according to the widely used CKD-EPI equation to estimate glomerular filtration rate (eGFR) from serum creatinine as follows [12]:

$$ \mathrm{GFR}=141\ast \min\ {\left(\mathrm{Scr}/\upkappa, 1\right)}^{\upalpha}\ast \max {\left(\mathrm{Scr}/\upkappa, 1\right)}^{\hbox{-} 1.209}\ast 0{.993}^{\mathrm{Age}}\ast 1.018\left[\mathrm{if}\ \mathrm{female}\right]\ast 1.159\left[\mathrm{if}\kern0.5em \mathrm{black}\right] $$

Scr is serum creatinine (mg/dL), *κ* is 0.7 for females and 0.9 for males, *α* is − 0.329 for females and − 0.411 for males, min indicates the minimum of Scr/κ or 1 and max indicates the maximum of Scr/*κ* or 1.

The five stages of CKD and eGFR for each stage were stage 1 with eGFR > 90 mL/min/1.73m^2^, stage 2 with an eGFR of 60–89 mL/min/1.73m^2^, stage 3A with an eGFR of 45–59 mL/min/1.73m^2^, stage 3B with an eGFR of 30–44 mL/min/1.73m^2^, stage 4 with an eGFR of 15–29 mL/min/1.73m^2^ and stage 5 with an eGFR < 15 mL/min/1.73m^2^ or receiving dialysis or renal transplantation.

### Confounding variables

We included confounding covariates that were likely to be associated with the risk of a deterioration in renal function. These included age at the diagnosis of gout, gender, Charlson comorbidity index, other comorbidities and medications. The Charlson comorbidity index summarizes 17 diagnostic categories (myocardial infarction, congestive heart failure, peripheral vascular disease, cerebrovascular disease, dementia, chronic pulmonary disease, rheumatologic disease, peptic ulcer disease, mild liver disease, moderate or severe liver disease, diabetes mellitus, diabetes mellitus with chronic complications, renal diseases, any malignancy [including leukaemia and lymphoma], metastatic solid tumours and human immunodeficiency virus infection) to represent health status, and it has been shown to be a useful predictor of mortality [13]. Deyo et al. produced a validated version for use with International Classification of Diseases version 9 ICD-9-based databases [14], and we used this version to calculate the index. Medications considered in this study included anti-hypertensive drugs, NSAIDs, insulin, oral hypoglycaemic agents, lipid-lowering agents, aspirin, glucocorticoid and colchicine. NSAIDs and glucocorticoid are mostly in as-needed dose. Laboratory covariates included serum uric acid levels and baseline eGFR.

### Statistical analysis

The incidence of renal function impairment was calculated using the number of people with CKD progression by one stage after the index date as the numerator and the person-days of the gout patients with different stages of CKD at the index date as the denominators.

We conducted three different analyses to assess the association between ULD use and CKD progression, recovery or improvement. The first analysis used propensity score (PS) weighting for multiple ULDs [15] and cause-specific Cox proportional hazards model to estimate the hazard ratios (HRs) and 95% confidence intervals (CIs) adjusted for aforementioned covariates [16]. Proportionality assumption was examined by the log-log plot. The original cohort of 5860 people was analysed. The PS weighting was conducted using the TWANG package, considering the aforementioned covariates [17]. Inverse probability of treatment weights of propensity scores was used to balance covariates across the ULD groups. The weights for optimal balance were estimated using generalized boosted models with 5000 regression trees.

The second approach used the Cox proportional hazards model to estimate the association between ULDs and CKD progression in the original cohort of 5860 patients. Among patients with CKD progression, we conducted a separate analysis to estimate the incidence of CKD recovery. Cox proportional hazard model were used to estimate HRs (95% CIs) for CKD recovery adjusted for aforementioned covariates which were identified at the time of CKD progression. All statistical analyses were performed using SAS statistical software, V.9.4.

## Results

The source population included 30,077 patients with a diagnosis of gout between 2012 and 2015. Our analysis included 5860 gout patients who exposed to one ULD (Fig. 1). The mean age of the patients at the index date was 61.09 ± 15.29 years, and 78.52% were male. Table 1 shows the baseline characteristics at the index date. Overall, there were 1329 patients (22.68%) with CKD stage 1, 1936 (33.04%) with CKD stage 2, 2018 (34.44%) with CKD stage 3 and 577 (9.85%) with CKD stage 4. The median time from the initial registration date to the index date was 8 years (interquartile range 2–12 years).

**Table 1:** Baseline characteristics

	Entire cohort (*n* = 5860)		Type of medication						*P* value
			Allopurinol (*n* = 532)		Febuxostat (*n* = 1105)		Other uricosuric agents (*n* = 4223)		
	*n*	%	*n*	%	*n*	%	*n*	%	
Age (years) (mean ± SD)	61.09 ± 15.29		61.68 ± 14.95		63.91 ± 15.00		60.28 ± 15.32		< .0001
Sex									0.0623
Female	1259	(21.48)	94	(17.67)	233	(21.09)	932	(22.07)	
Male	4601	(78.52)	438	(82.33)	872	(78.91)	3291	(77.93)	
CKD stage									< .0001
CKD stage 1	1329	(22.68)	113	(21.24)	85	(7.69)	1131	(26.78)	
CKD stage 2	1936	(33.04)	163	(30.64)	147	(13.30)	1626	(38.50)	
CKD stage 3	2018	(34.44)	204	(38.35)	557	(50.41)	1257	(29.77)	
CKD stage 4	577	(9.85)	52	(9.77)	316	(28.60)	209	(4.95)	
eGFR (mm/min/1.37 m^2^) (mean ± SD)	66.74 ± 28.63.		65.10 ± 28.39		45.53 ± 24.42		72.49 ± 26.99		< .0001
CKD stage 1 (mean ± SD)	107.10 ± 12.91		107.96 ± 12.27		106.90 ± 13.21		107.02 ± 12.95		
CKD stage 2 (mean ± SD)	74.28 ± 8.48		73.04 ± 8.76		72.19 ± 8.45		74.59 ± 8.43		
CKD stage 3 (mean ± SD)	45.32 ± 8.54		45.56 ± 8.26		41.88 ± 8.10		46.81 ± 8.34		
CKD stage 4 (mean ± SD)	23.38 ± 3.97		24.03 ± 4.07		23.06 ± 4.07		23.71 ± 3.76		
Serum UA level (mg/dl) (mean ± SD)	9.49 ± 4.45		8.30 ± 3.74		9.92 ± 4.82		9.53 ± 4.40		< .0001
Comorbidity^†^									
Myocardial infarct	284	(4.85)	19	(3.57)	63	(5.70)	202	(4.78)	0.1602
Congestive heart failure	898	(15.32)	70	(13.16)	195	(17.65)	633	(14.99)	0.0320
Peripheral vascular disease	230	(3.92)	22	(4.14)	63	(5.70)	145	(3.43)	0.0025
Cerebrovascular disease	1021	(17.42)	100	(18.80)	190	(17.19)	731	(17.31)	0.6785
Diabetes mellitus	1760	(30.03)	191	(35.90)	412	(37.29)	1157	(27.40)	< .0001
Diabetes with chronic complications	713	(12.17)	85	(15.98)	220	(19.91)	408	(9.66)	< .0001
Cancers	636	(10.85)	54	(10.15)	139	(12.58)	443	(10.49)	0.1195
Metastatic solid tumour	92	(1.57)	6	(1.13)	22	(1.99)	64	(1.52)	0.3640
Hypertension	3892	(66.42)	377	(70.86)	819	(74.12)	2696	(63.84)	< .0001
Hyperlipidaemia	2587	(44.15)	235	(44.17)	469	(42.44)	1883	(44.59)	0.4414
Charlson comorbidity index (mean ± SD)	2.22 ± 2.18		2.48 ± 2.22		3.03 ± 2.46		1.98 ± 2.04		< .0001
Co-medication^‡^									
Anti-hypertensive drugs	2814	(48.02)	249	(46.80)	557	(50.41)	2008	(47.55)	0.2006
Diuretics	1582	(27.00)	135	(25.38)	399	(36.11)	1048	(24.82)	< .0001
Insulin	357	(6.09)	39	(7.33)	116	(10.50)	202	(4.78)	< .0001
Hypoglycaemic agents	1329	(22.68)	148	(27.82)	319	(28.87)	862	(20.41)	< .0001
Lipid lowering agents	1920	(32.76)	180	(33.83)	374	(33.85)	1366	(32.35)	0.5493
Aspirin	1299	(22.17)	117	(21.99)	244	(22.08)	938	(22.21)	0.9906
Colchicine	2733	(46.64)	261	(49.06)	480	(43.44)	1992	(47.17)	0.0433
Glucocorticoid*	1007	(17.27)	53	(9.96)	214	(19.37)	740	(17.52)	< 0.001
Non-steroidal anti-inflammatory drugs*	1974	(33.86)	112	(20.06)	231	(20.09)	1631	(38.62)	< 0.001

^†^Assessed before the first use of urate-lowering drug

^‡^Assessed during the 3 months before the first use of urate-lowering drug

*Dosing intervals were at ‘as-needed’ basis

During 434,176 person-days of follow-up, 741 events of CKD progression occurred (12.96%), which corresponds to an incidence rate (95% CI) of 1.70 (1.58–1.83) per 1000 person-days. The incidence rate (95% CI) for CKD progression was 1.98 (1.37–2.60) per 1000 person-days for allopurinol users, 1.88 (1.58–2.17) per 1000 person-days for febuxostat users and 1.64 (1.51–1.78) per 1000 person-days for uricosuric agent users. The 741 patients who had CKD progression were further followed for 158,861 person-days, of whom 571 (77.06%) had CKD recovery. The overall incidence (95% CI) of CKD recovery was 3.59 (3.30–3.89) per 1000 person-days, including 1.33 (0.82–1.84) per 1000 person-days for allopurinol users, 6.21 (5.11–7.31) per 1000 person-days for febuxostat users and 3.53 (3.20–3.87) per 1000 person-days for uricosuric agent users.

We conducted two different approaches to examine the CKD progression and improvement associated with ULD use. The first approach used the PS-weighted cause-specific Cox-proportional hazards models to examine the risk of CKD progression and improvement compared to the status in the index date in original cohort of 5860 patients (Table 2). Compared with allopurinol users, the PS-weighted HR (95% CI) for CKD progression was 1.77 (0.85–1.76) for febuxostat users and 1.37 (0.71–1.37) for uricosuric agent users. The PS-weighted HR (95% CI) for CKD improvement was 1.43 (1.26–1.62) for febuxostat users and 1.00 (0.88–1.14) for uricosuric agent users.

**Table 2:** PS-weighted cause-specific Cox proportional hazard regression to estimate hazard ratio for renal function deterioration or improvement by baseline CKD stage using allopurinol as reference

	Hazard ratio (95% CI)	
	Renal function deterioration	Renal function improvement
Entire cohort	–	–
Type of ULD		
Allopurinol	Reference	Reference
Febuxostat	1.77 (0.85–1.76)	1.43 (1.26–1.62)*
Uricosuric agents	1.37 (0.71–1.37)	1.00 (0.88–1.14)
eGFR ≥ 90		
Allopurinol	Reference	–
Febuxostat	1.97 (0.95–4.08)	–
Uricosuric agents	0.91 (0.48–1.70)	–
eGFR 60–89		
Allopurinol	Reference	Reference
Febuxostat	1.49 (0.74–3.02)	1.88 (1.44–2.45)*
Uricosuric agents	1.15 (0.63–2.08)	1.33 (1.02–1.72)*
eGFR 30–59		
Allopurinol	Reference	Reference
Febuxostat	0.96 (0.53–1.74)	1.12 (0.84–1.49)
Uricosuric agents	0.93 (0.52–1.67)	0.95 (0.72–1.26)
eGFR 15–29		
Allopurinol	Reference	Reference
Febuxostat	1.49 (0.43–5.15)	1.32 (0.80–2.20)
Uricosuric agents	1.39 (0.40–4.82)	1.46 (0.88–2.42)

* *p* < 0.05

Secondly, we compared CKD progression for the original cohort and CKD recovery for the patients who had CKD progression in the first place. Cox proportional hazards models were used to assess the association. Compared with allopurinol users, the adjusted HR (95% CI) for CKD progression was 1.14 (0.80–1.66) for febuxostat users and 0.92 (0.67–1.31) for uricosuric agent users. We conducted stratified analysis according to baseline CKD stage and found similar findings as in the main analysis (Table 3).

**Table 3:** Incidence of renal function deterioration and adjusted hazard ratio for renal function deterioration by baseline CKD stage using allopurinol as reference

	Person-days of follow-up	Number of patients	Events of CKD progression	Incidence rate (95% CI) per 1000 person-days	Hazard ratio (95% CI)
Entire cohort	434,716	5860	741	1.70 (1.58–1.83)	–
Type of ULD					
Allopurinol	19,680	532	39	1.98 (1.36–2.60)	Reference
Febuxostat	83,022	1105	156	1.88 (1.58–2.17)	1.14 (0.80–1.66)
Uricosuric agents	332,014	4223	546	1.64 (1.51–1.78)	0.92 (0.67–1.31)
eGFR ≥ 90					
Allopurinol	4031	113	11	2.73 (1.12–4.34)	Reference
Febuxostat	5861	85	27	4.61 (2.87–6.34)	1.99 (0.99–4.28)
Uricosuric agents	97,969	1131	208	2.12 (1.83–2.41)	0.91 (0.51–1.82)
eGFR 60–89					
Allopurinol	6134	163	12	1.96 (0.85–3.06)	Reference
Febuxostat	9141	147	25	2.73 (1.66–3.81)	1.43 (0.72–2.99)
Uricosuric agents	123,774	1626	225	1.82 (1.58–2.06)	1.12 (0.64–2.14)
eGFR 30–59					
Allopurinol	7873	204	14	1.78 (0.85–2.71)	Reference
Febuxostat	44,744	557	76	1.70 (1.32–2.08)	0.97 (0.55–1.84)
Uricosuric agents	95,166	1257	97	1.02 (0.82–1.22)	0.93 (0.53–1.75)
eGFR 15–29					
Allopurinol	1642	52	2	1.22 (0.15–4.40)	Reference
Febuxostat	23,276	316	28	1.20 (0.76–1.65)	1.40 (0.33–10.15)
Uricosuric agents	15,105	209	16	1.06 (0.54–1.58)	1.28 (0.30–9.34)

Compared with allopurinol exposure, the HR (95% CI) for CKD recovery was 2.17 (1.40–3.47) for febuxostat and 1.80 (1.20–2.83) for uricosuric agent exposure. Table 3 shows a stratified analysis. Compared with allopurinol users, febuxostat and uricosuric agent users were more likely to recover one CKD stage among those with CKD stages 3 and 4 (Table 4).

**Table 4:** Renal function recovery after the 1st date of renal function decline among patients with renal function deterioration (*n* = 760)

	Person-days of follow-up	Number of patients	Events of CKD recovery	Incidence rate (95% CI) per 1000 person-days	Hazard ratio (95% CI)
Entire cohort	158,861	741	571	3.59 (3.30–3.89)	–
Type of ULD					
Allopurinol	19,509	39	26	1.33 (0.82–1.84)	Reference
Febuxostat	19,639	156	122	6.21 (5.11–7.31)	2.17 (1.40–3.47)*
Uricosuric agents	119,713	546	423	3.53 (3.20–3.87)	1.80 (1.20–2.83)*
eGFR 60–89					
Allopurinol	3896	11	9	2.31 (1.06–4.39)	Reference
Febuxostat	5390	24	19	3.53 (1.94–5.11)	0.88 (0.39–2.13)
Uricosuric agents	54,434	202	148	2.72 (2.28–3.16)	0.87 (0.44–1.91)
eGFR 30–59					
Allopurinol	5615	12	8	1.42 (0.62–2.81)	Reference
Febuxostat	2866	26	21	7.33 (4.19–10.46)	2.69 (1.18–6.71)*
Uricosuric agents	46,123	216	167	3.62 (3.07–4.17)	2.18 (1.10–4.99)*
eGFR 15–29					
Allopurinol	7004	14	9	1.28 (0.59–2.44)	Reference
Febuxostat	7913	76	62	7.84 (5.88–9.79)	2.29 (1.03–5.65)*
Uricosuric agents	16,598	103	87	5.24 (4.14–6.34)	1.98 (0.91–4.75)
eGFR < 15					
Allopurinol	2994	2	0	– –	Reference
Febuxostat	3470	30	20	5.76 (3.24–8.29)	–
Uricosuric agents	2558	25	21	8.21 (4.70–11.72)	–

The eGFR groups were based on the value of the first date of renal function decline, and the outcome was an improvement in renal function to more than one eGFR group

* *p* < 0.05

## Discussion

In this study using data from a large cohort of gout, one in nine patients exposed to ULDs had CKD progression irrespective of the urate-lowering mechanism of action. Gout patients were at a high risk of renal deterioration even for those with a relatively preserved renal function probably due to their concomitant risk factors such as renal toxic medication and comorbidities. The risk of CKD progression was similar among those exposed to allopurinol, febuxostat and uricosuric agents, and this effect was consistent across the patients with different stages of CKD at baseline. Furthermore, CKD recovery was also common among patients with CKD progression, among whom approximately three quarters had improvement in renal function. However, compared to the patients receiving allopurinol, those receiving febuxostat and uricosuric agents had a two-fold higher likelihood of recovery. To the best of our knowledge, this is the first study to examine the risk of CKD progression and recovery in patients with gout after exposure to different classes of ULDs.

Human studies have provided limited evidence of a link between hyperuricaemia and deterioration in renal function. One early study found that mild asymptomatic hyperuricaemia was associated with decreased renal blood flow [18]. A more recent study found that patients with the highest quintile of serum uric acid level, compared to those with the lowest quintile, had an odds ratio of 1.49 for renal function deterioration [19]. Another study found that an increase of 2 mg/dL in serum uric acid level was associated with an odds ratio of 1.69 for CKD [20]. Moreover, patients with hyperuricaemia have been reported to be more likely to develop end-stage renal disease [21]. In our previous study, patients with hyperuricaemia were associated with an annual decline in eGFR by 2.5 mL/min/1.73 m^2^, almost twice the rate of decline noted in the normouricaemia group [22]. Therefore, it seems reasonable that ULDs may halt the progression of CKD in patients with gout.

Limited evidence supports allopurinol as a renal protective agent. A recent real-world propensity score-matched cohort study found that allopurinol initiators were associated with an HR of 0.87 (95% CI, 0.77–0.97) for developing CKD stage 3 or higher [23]. A small clinical trial randomized 54 hyperuricaemic patients with CKD to 100–300 mg/day of allopurinol or usual care and found no statistical difference in serum creatine level after 12 months of therapy but far fewer renal endpoints (a 40% increase in serum creatinine or dialysis) [24]. Another clinical trial recruited 113 participants with a mean eGFR of 40 mL/min/1.73 m2 and randomized them into allopurinol or usual care groups. After 84 months of follow-up, 7 of 57 allopurinol users and 24 of 56 controls met renal endpoints (a twofold increase in creatinine or dialysis) [25]. However, these trials were small and focused on people who already had CKD. Recent larger clinical trials comparing allopurinol and febuxostat seem to support that febuxostat has a greater renal protective effect. A recent meta-analysis summarized the results from four clinical trials and found that the mean difference in changes in eGFR at 1 month after treatment was 1.65 mL/min/1.73 m^2^ between groups which favoured febuxostat, although the changes were not significantly different at 3 months [26].

Our main results from routine clinical care are supported by another recent study from Taiwan, which found that among CKD patients, allopurinol users were more likely to need dialysis [27]. However, there are important differences between the current and previous studies which may have affected the interpretation of the results. First, our study is an observational study based on routine care, and therefore, our results reflect real-world experience. In addition, our outcome definitions allowed us to determine the risk of CKD progression and recovery. Second, the larger sample size meant that our comparisons could be conducted in each CKD stage. Considering these differences, it seems reasonable to conclude that CKD progression and recovery are common in patients with gout after exposure to ULDs and that febuxostat and uricosuric agents are better than allopurinol in terms of renal function, particularly in those with existing CKD.

### Limitations

There are several limitations to this study. First, misclassification may exist since the identification of the patients with gout was made by physician-based diagnoses. The patient cohort was from a medical center which tends to be more severe. Second, residual confounding may exist, for example with respect to a physical activity for which data were not available. Third, we only considered ULD prescriptions rather than actual patient consumption, and gout patients are known to have the worst drug adherence of all patients with chronic illnesses. We also did not consider ULD doses into consideration [28]. Fourth, the classification of CKD, its progression and recovery were based on eGFR at the time of examination. Some temporary conditions such as dehydration and exposure to nephrotoxic agents are reversible and not necessarily predispose to chronicity. Therefore, our data cannot represent the effect of ULD exposures on the chronicity of CKD, rather, should be acute and reversible renal injury. Fifth, some patients are not followed long enough to identify renal function deterioration since CKD generally progresses slowly. Sixth, gout characteristics such as tophi are not well and consistently recorded in diagnosis codes. Therefore, we cannot adjust for these potential confounders. Seventh, we excluded patients without regular follow-up and those without ULD use. Only 5882 patients were included. Therefore, a selection bias toward more compliant patients may occur. Eighth, fixed-duration assessment period between the initial registration date and the index date may introduce bias [29], despite the median length of the period was long (8 years). Therefore, the risk of introducing this bias is small. Finally, collider bias may arise and lead to paradoxical association between ULD use and the CKD recovery [30]. However, we aim to show that both CKD progression and recovery are common in gout patient who exposed to ULD. The patients have high prevalence of comorbidities and co-medications. The effect of ULD on patients with an eGFR > 90 ml/min/m^2^ is difficult to measure in this context.

## Conclusions

CKD progression in gout patients was not an infrequent observation after the initiation of ULDs and renal function recovery was observed in three quarters of those who had CKD progression. CKD progression was less common in patients exposed to febuxostat and uricosuric agents compared to those exposed to allopurinol, particularly for those with existing CKD.

## Data Availability

Please contact the author for data requests.

## Abbreviations

CKD: Chronic kidney disease
HRs: Hazard ratios
CIs: Confidence intervals
ULDs: Urate-lowering drugs
NSAIDs: Non-steroidal anti-inflammatory drugs
